# Expanding the chemical information science gateway

**DOI:** 10.12688/f1000research.12772.1

**Published:** 2017-09-27

**Authors:** Jürgen Bajorath

**Affiliations:** 1 Department of Life Science Informatics, B-IT, LIMES Program Unit Chemical Biology and Medicinal Chemistry, Rheinische Friedrich-Wilhelms-Universität, Bonn, Germany

**Keywords:** chemical information science, chemoinformatics, experimental chemistry, interdisciplinary research, drug discovery, big data, open peer review, publishing, data sharing

## Abstract

Broadly defined, chemical information science (CIS) covers chemical structure and data analysis including biological activity data as well as processing, organization, and retrieval of any form of chemical information. The CIS Gateway (CISG) of F1000Research was created to communicate research involving the entire spectrum of chemical information, including chem(o)informatics. CISG provides a forum for high-quality publications and a meaningful alternative to conventional journals. This gateway is supported by leading experts in the field recognizing the need for open science and a flexible publication platform enabling off-the-beaten path contributions. This editorial aims to further rationalize the scope of CISG, position it within its scientific environment, and open it up to a wider audience. Chemical information science is an interdisciplinary field with high potential to interface with experimental work.

## Chemical information science

Chemical information science (CIS) comprises any form of chemical structure or data analysis and chemical information retrieval ^1^. Chem(o)informatics, which organizes and extracts knowledge from functional data associated with small molecules, analyzes structure-activity relationships, and predicts active compounds, is considered to be a part of CIS ^1, 2^. In addition, CIS encompasses ‘big data’ analysis in chemistry, which is an emerging field ^3^. Hence, CIS represents a wide spectrum of computational efforts and also interfaces with experimental chemistry, especially synthetic and medicinal chemistry, and interdisciplinary drug discovery research. The derivation of experimentally testable hypotheses, design of experiments, and large-scale analysis of experimental data are among the central tasks of CIS, in addition to information retrieval from chemical data and the development of computational methods and infrastructures.

## CIS gateway and advisory board

The CIS Gateway (CISG) of F1000Research was conceptualized and initiated in 2015 to provide a venue for open access publication of high-quality papers ^2^ with open post-publication peer review ^4^. It has been the first –and continues to be the only– open review publication platform for CIS including chem(o)informatics.

To put emphasis on high-quality publications, CISG has assembled an international advisory board of leading experts and implemented a two-stage review process that is coordinated by gateway advisors. Submissions to CISG are first forwarded to the advisory board to conduct a pre-review (stage 1) and assess whether or not a manuscript fits the scope of CISG and has scientific potential to advance the field. This assessment does not restrict the type of contributions that are submitted, as further discussed below, but serves as an initial checkpoint for scientific quality. Conclusions of the advisory board are forwarded to the authors. If a manuscript is considered to be relevant and of sufficient scientific potential, it is published in CISG and subjected to open peer review (stage 2); if not, authors still have the option to publish their work in F1000Research outside CISG. Members of the advisory board help with reviewer suggestions for CISG publications and frequently participate in the open peer review process.

## Scope

CISG is open to submission of the entire portfolio of F1000Research manuscript categories, except clinical practice articles and editorials (by invitation only). Original research and methods articles, data notes, and software tool descriptions are encouraged and so are opinion articles. The latter offer investigators the opportunity to provide personal accounts of CIS-related topics, articulate their views, and initiate open discussions in the field utilizing the CISG platform. Moreover, CISG strongly encourages off-the-beaten-path contributions including the publication of negative results, demonstration of methodological failures or conceptual flaws, and discussion of new or visionary CIS concepts, even if perceived controversially.

Also welcome are case studies presenting individual projects. Case studies provide excellent opportunities to report on interdisciplinary projects combining computational and experimental approaches, for example, in drug discovery research, evaluate computational predictions, and showcase the interplay between computations and experiments.

Although CISG has been devised in the spirit of open science and data sharing, it is well understood that researchers from the pharmaceutical or other industries usually are not at liberty to disclose their data such as structures of novel chemical entities. This should not prevent the publication of scientifically stimulating case studies or other contributions in CISG. It is a strength of CISG’s two-stage review process that partly confidential studies can be evaluated by the advisory board and approved for publication if they represent compelling scientific insights and stories, even if not all the data reported can be freely shared.

The advent of the ‘big data’ era in chemistry offers additional opportunities. Rapidly growing volumes of increasingly complex and heterogeneous chemical data challenge database structures as much as data analysis and knowledge extraction ^3^. New methodological developments are required to handle big data and data note publications communicating related issues will play an important role going forward.

Among others, current CISG publications include research articles describing the analysis of biologically relevant molecular similarity values ^5^ or introducing a mathematical framework for the study of polypharmacology ^6^ and data notes reporting curated sets of kinase inhibitors ^7^ for computational research or the collection of all two- and three-dimensional activity cliffs that could be isolated from public chemical structure and activity data sources ^8^. CISG already contains publications covering rather diverse research topics and it is sincerely hoped that there will be more to come.

In summary, CISG offers a flexible publication platform for an unprecedentedly diverse array of contributions that should be attractive to many investigators in the field.

## Competitive landscape

CISG is positioned in a core area where it competes with conventional journals such as the Journal of Chemical Information and Modeling (J. Chem. Inf. Model.; American Chemical Society), Journal of Cheminformatics (J. Cheminf.; Chemistry Central), or Molecular Informatics (Mol. Inf.; Wiley). Among these, CISG is the only open peer review publication platform and hence fills a void. In addition, J. Chemif. and CISG are open access publications. Historically, J. Chem. Inf. Model. and its precursor, the Journal of Chemical Information and Computer Sciences (J. Chem. Inf. Comput. Sci.) have been flagship journals for the CIS and chem(o)informatics community. However, over the past few years, J. Chem. Inf. Model. has been changing its focus, departing from CIS, and increasingly concentrating on molecular simulations and modeling. As a consequence, currently there is no longer a clear hierarchy among conventional journals in the CIS arena and new opportunities for publication of CIS research are desirable. Clearly, the possibility to accommodate off-the-beaten-path contributions, which CISG strongly encourages, sets it apart from conventional journals and provides further motivation to consider CISG for dissemination of first-class research. Efficient processing of submissions and much shorter publication times of CISG are additional benefits.

## Audience

CIS itself is an interdisciplinary field that spans a wide range of computational sub-disciplines, as discussed above. However, there are ample opportunities to further increase its interdisciplinary nature by exploring interfaces of CIS with experimental work involving chemistry. A good example is the still evolving field of chemical biology, which benefits from the integration of computational approaches ^9^. Hence, CISG should also be of considerable interest to a wider scientific community beyond computational experts. This expansion is further supported by the diversity of contributions CISG is able to accommodate, as described above.

## The future

CISG strives for publishing top-level research including method development and applications and will put further emphasis on off-the-beaten-path contributions, which set it apart from conventional journals. The ultimate goal of CISG is becoming a primary venue for publication of research in the broadly defined area of CIS, also involving interdisciplinary work with experimental components from the life sciences. Each CISG publication should be regarded as a must-read in the field, an ambitious but feasible goal. Last but not least, a noted strength of CISG is support by leading investigators recognizing the need for open science. Going forward, this culture will be fostered, focusing on contributions from a premier advisory board.
